# Supplementary material for: Antitumor Activity of USP7 Inhibitor GNE-6776 in Non-Small Cell Lung Cancer Involves Regulation of Epithelial-Mesenchymal Transition, Cell Cycle, Wnt/β-Catenin, and PI3K/AKT/mTOR Pathways
Source: Pharmaceuticals (Basel). 2025 Feb 12;18(2):245. doi: 10.3390/ph18020245 (PMC11858873; doi:10.3390/ph18020245)
Supplement: Supplementary file 1 [file pharmaceuticals-18-00245-s001.zip › Supplementary material Figure S1 Identification of USP7 as a Driver of NSCLC Oncogenesis.pdf]

Supplementary material Figure S1: Identification of USP7 as a Driver of NSCLC Oncogenesis

Methods

DepMap, a database using RNAi and CRISPR-Cas9 technology to screen potential therapeutic targets in cancer, provides gene dependency data for various cancer cell lines. CRISPR-Cas9 cleavage can cause severe DNA damage, leading to apoptosis and growth arrest, which may result in false positive results. Considering sgRNA dropout and gene copy numbers, DepMap instead uses the parameter, “CERES score,” to measure gene essentiality. The principle of the CERES score is that the fewer the cells that survive with the gene’s sgRNA and the fewer the copies of the gene in the cells, the stronger the dependency on the gene. A negative CERES score implies that knockout of the gene inhibits cell proliferation and survival, with a lower score indicating greater gene importance. Additionally, the Xena database was used to analyze USP7 expression differences between normal tissue and lung cancer tissue, and the HPA database was used to explore USP7 expression in NSCLC.

Rsults

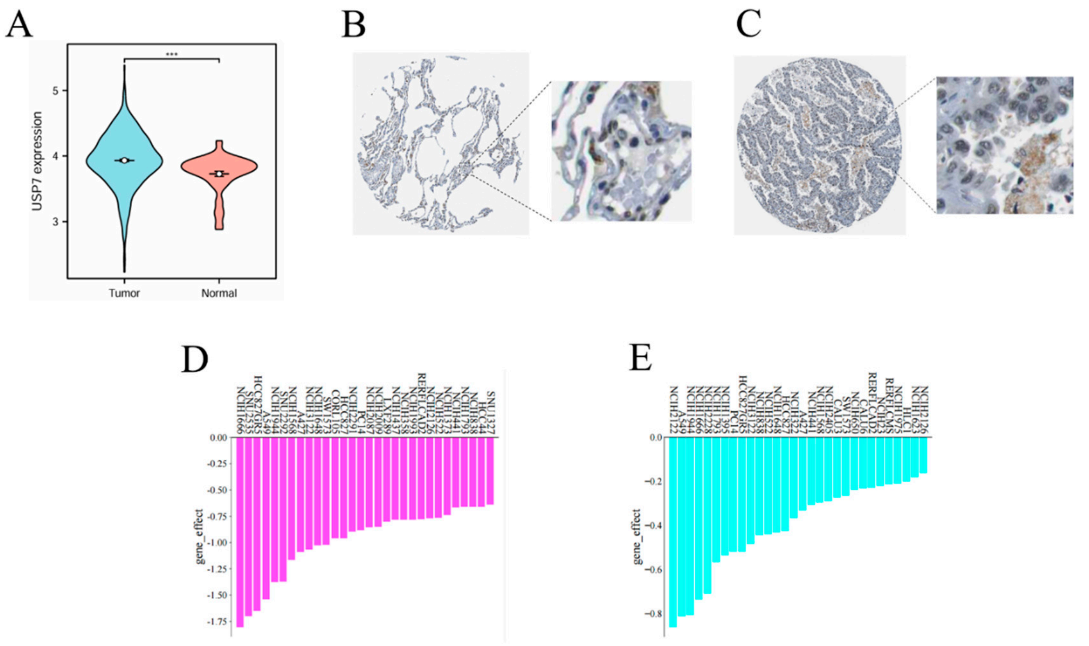

Figure S1. Identification of USP7 as a Driver of NSCLC Oncogenesis

Analysis of the essential role of USP7 in non-small cell lung cancer (NSCLC) cell viability demonstrated by the USP7 dependency of non-small cell lung cancer cell lines using the DepMap dataset and by USP7 knockdown via RNAi and CRISPR-Cas9. Additionally, the protein expression of USP7 in normal lung

tissue versus lung adenocarcinoma tissue from the HPA database is shown.

(A) Validation of USP7 mRNA expression in NSCLC versus normal lung tissue using public datasets. (B) Immunohistochemistry (IHC) staining of USP7 protein expression in normal lung tissue. (C) IHC staining of USP7 protein expression in lung adenocarcinoma tissue. (D) RNAi-mediated knockdown of USP7 in NSCLC cells. (E) CRISPR-Cas9-mediated knockout of USP7.
