# Supplementary material for: Antitumor Activity of USP7 Inhibitor GNE-6776 in Non-Small Cell Lung Cancer Involves Regulation of Epithelial-Mesenchymal Transition, Cell Cycle, Wnt/β-Catenin, and PI3K/AKT/mTOR Pathways
Source: Pharmaceuticals (Basel). 2025 Feb 12;18(2):245. doi: 10.3390/ph18020245 (PMC11858873; doi:10.3390/ph18020245)
Supplement: Supplementary file 1 [file pharmaceuticals-18-00245-s001.zip › Supplementary material Figure S2 Effect of GNE-6776 on the proliferation of Beas-2B cells.pdf]

Supplementary material Figure S2. Effect of Gne-6776 on the proliferation of Beas-2B cells

Methods

Cell Culture

Beas-2B cells were cultured in RPMI 1640 medium (Gibco, USA) supplemented with 10% fetal bovine serum (Vivacell, China) and 1% penicillin-streptomycin (Gibco, USA) at 37°C in a 5% CO_2 atmosphere. The cells were passaged regularly.

Results

The results indicated that Gne-6776 had no noticeable effects on normal human lung epithelial cells (Beas-2B), even at a concentration of 100 μM, and therefore exhibited low inhibitory activity against normal cells (Figure S2).

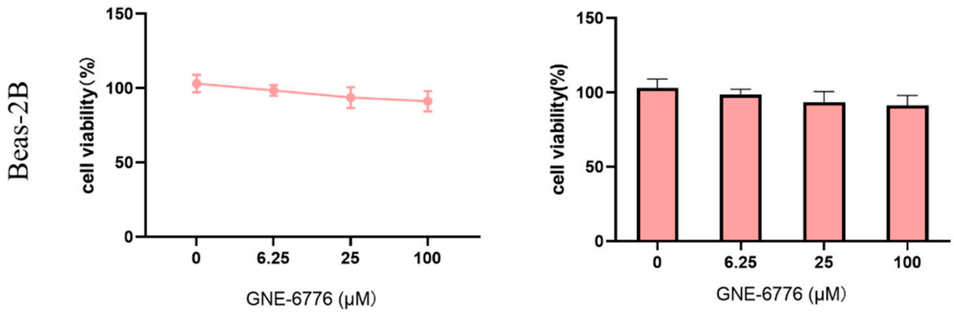

Figure S2. Effect of Gne-6776 on the proliferation of Beas-2B cells.
Beas-2B cells were treated with increasing concentrations of Gne-6776 (0, 6.25, 25, 100 μM) for 24 h. Cell viability was measured using a CCK-8 assay. Data are presented as mean ± SEM (n = 9). Statistical significance was analyzed using one-way ANOVA (* $p < 0.05$, ** $p < 0.01$).
