# Supplementary material for: Antitumor Activity of USP7 Inhibitor GNE-6776 in Non-Small Cell Lung Cancer Involves Regulation of Epithelial-Mesenchymal Transition, Cell Cycle, Wnt/β-Catenin, and PI3K/AKT/mTOR Pathways
Source: Pharmaceuticals (Basel). 2025 Feb 12;18(2):245. doi: 10.3390/ph18020245 (PMC11858873; doi:10.3390/ph18020245)
Supplement: Supplementary file 1 [file pharmaceuticals-18-00245-s001.zip › Supplementary material Figure S3 Histopathological analysis of the tumor tissue by HE staining.pdf]

Supplementary material Figure S3: Histopathological analysis of the tumor tissue by HE staining

Methods

Observation of Histopathological Morphological Changes in Tumor Tissue Using HE Staining

The tumor tissue was fixed with 4% paraformaldehyde, dehydrated, and cleared with ethanol and xylene. Tissue was then immersed in wax for 1 h at 58°C in three successive wax baths. The tissue was placed in embedding molds, which were then positioned in a metal frame and filled with paraffin that was then allowed to solidify. The embedded wax blocks containing the tissue were trimmed to a flat surface to fully expose the tissue. Subsequently, the blocks were sectioned, and the sections were mounted on slides, dewaxed, and stained with hematoxylin and eosin (HE). After re-dehydration and clearing, the tissue was mounted on slides with coverslips and observed using a panoramic Scanner(Leica, Hessen, Germany).

Results

In the control group, the tumor cells were arranged in a neat and dense manner, with increased nuclear chromatin, with no significant cell necrosis observed. In the Gne-6776 low-concentration group, the density of the tumor cell arrangement decreased, the increased nuclear chromatin observed in the control group was slightly reduced, and a certain degree of cell necrosis was present. In both the Gne-6776 high-concentration group and the DDP group, the cells were arranged sparsely, the increased nuclear chromatin was significantly reduced, and the degree of necrosis markedly increased (Figure S3).

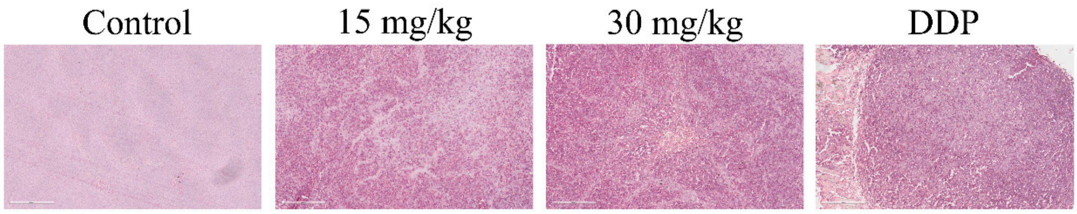

Figure S3. Histopathological analysis of the tumor tissue by HE staining.

Representative images of HE-stained tumor sections from nude mice treated with the vehicle control, Gne-6776, or DDP. Scale bar: 200 μm; magnification: ×200.
